# Chemical Antidote for Hydrocyanic Acid

**Published:** 1845-08-01

**Authors:** 


*Chemical Antidote for Hydrocyanic Acid.*—Messrs. J. and J. H. Smith, of Edinburgh, have shown that "the effects of a fatal dose of Prussic acid may be warded off by the timely administration of the re-agents necessary for converting the acid into Prussian blue. They found that if a solution of carbonate of potash, followed by a solution of the mixed sulphates of iron, be given to animals very soon after the administration of thirty drops of the Edinburg medicinal acid, containing 3 per cent. real acid, recovery in general takes place, and sometimes but little inconvenience seems to be sustained. The solutions they used were one of 144 gr. carb. pot. in 2 scr. of water, and another composed of gr. iss. sulph. protox. iron, together with gr. ii. of the same salt converted in sulph. of sesquioxide, by means of sulphuric and nitric acids, in the usual way. About 52 minims of each solution will remove the whole of the acid contained in 100 gr. of Ed. medicinal acid; but for certainty, three or four times as much should be used—which may be done with perfect safety." The above is an extract from the late edition of the treatise on poisons by Dr. Christison, which is quoted in the Medico Chirurg. Review for April, 1845. In the reprint of the London Lancet for January, 1845, may be found a communication from the Messrs. Smiths, giving a detailed account of their experiments, with more minute directions for the preparation of the antidote, and a discussion of the chemical principles upon which it is based.

